# Psychometric Properties of the Arabic Big Five Inventory-2 Short Form among Undergraduates in Kuwait

**DOI:** 10.1192/j.eurpsy.2022.1718

**Published:** 2022-09-01

**Authors:** B. Alansari, T. Alali

**Affiliations:** Kuwait University, College of Social Sciences, Psychology Department, Kaifan, Kuwait

**Keywords:** Psychometric Properties ,BFI-2 Short

## Abstract

**Introduction:**

The BFI-2-S assesses the domain level of the Big Five with three prototypical facets of each domain capturing approximately 91% of the total variance in the full BFI-2 domain scales and approximately 89% of the predictive power of the BFI-2 facets in German adaptations and their original American versions.

**Objectives:**

The study aims to investigate the psychometric properties of the Arabic adaptation of the BFI-2 short form.

**Methods:**

The Arabic version of the BFI-2-S a 30-item with 15 and NEO Five-Factor Inventory (NEO–PI-R) were administered to 1560 (576 males, 984 females) Kuwait University undergraduates with a mean age = 22.75 ± 3.81. The internal consistency reliability, factor structure, and convergent validity of the BFI-2-S with NEO–PI-R were assessed.

**Results:**

Cronbach’s alpha was satisfactory for N (0.79), E (0.73), O (0.73), A (0.76) and C (0.77). Results revealed significant gender differences in O, C & E with a favor for males and in N a favor with females. PCA showed that BFI-2-S five factors explains 64.38% of the total variance. However, the high mean correlations between the BFI-2-S and NEO–PI-R scales, with coefficients of (0.67) for the N, (0.66) for the E, (0.56) for the C, (0.61) for the A, and (0.58) for the C. The convergence between each BFI-2-S domain correlated substantially with the relevant NEO-PI-R domain scales, with the average correlation being .62.

**Conclusions:**

The findings support the psychometric properties of the Arabic adaptations of the BFI-2-S as useful instruments for assessing the Big Five.

**Disclosure:**

No significant relationships.

